# The place and role of (moral) anger in organizational behavior studies

**DOI:** 10.1002/job.2065

**Published:** 2015-12-07

**Authors:** Dirk Lindebaum, Deanna Geddes

**Affiliations:** ^1^ The University of Liverpool Management SchoolLiverpoolU.K.; ^2^ Fox School of BusinessTemple UniversityPhiladelphiaPennsylvaniaU.S.A.

**Keywords:** anger, aggression, indignation, morality, moral anger, revenge, righteous anger, moral outrage, empathic anger, personal anger, whistle‐blowing, injustice, conflict, emotion

## Abstract

The aim of this article is to conceptually delineate moral anger from other related constructs. Drawing upon social functional accounts of anger, we contend that distilling the finer nuances of morally motivated anger and its expression can increase the precision with which we examine prosocial forms of anger (e.g., redressing injustice), in general, and moral anger, in particular. Without this differentiation, we assert that (i) moral anger remains theoretically elusive, (ii) that this thwarts our ability to methodologically capture the unique variance moral anger can explain in important work outcomes, and that (iii) this can promote ill‐informed organizational policies and practice. We offer a four‐factor definition of moral anger and demonstrate the utility of this characterization as a distinct construct with application for workplace phenomena such as, but not limited to, whistle‐blowing. Next, we outline a future research agenda, including how to operationalize the construct and address issues of construct, discriminant, and convergent validity. Finally, we argue for greater appreciation of anger's prosocial functions and concomitant understanding that many anger displays can be justified and *lack* harmful intent. If allowed and addressed with interest and concern, these emotional displays can lead to improved organizational practice. © 2015 The Authors Journal of Organizational Behavior Published by John Wiley & Sons Ltd.

Don't make me angry, you won't like it when I'm angry.
(Bruce Banner, in *The Incredible Hulk*)
Anger is antisocial, unpleasant, negative, and very common
(Kemp & Strongman, 1995, p. 407)

## Introduction

Anger regularly gets bad press, and the earlier quotes are a clear testament to this effect. Anger is generically defined as “a strong feeling of displeasure and usually of antagonism” (Merriam‐Webster Online Dictionary, 2008). Consequently, it is not difficult to understand why anger is often provided as a prime example of “negative” emotion (Barclay, Skarlicki, & Pugh, 2005; Bodenhausen, Sheppard, & Kramer, 1994; Tiedens, 2001; Waldman, Balthazard, & Peterson, 2011). Anger is associated with an exhaustive list of adverse if not destructive consequences in the workplace and beyond, ranging from aggression, violence, and bullying (Chen & Spector, 1992; Matthiesen & Einarsen, 2007; Novaco, 1994) to ineffective leadership (Waldman et al., 2011) and deviance (Geddes & Callister, 2007; Geddes & Stickney, 2011). Indeed, many descriptions of anger circulating in psychological and management studies reflect this squarely. For instance, many psychologists associate the expression of anger “with *an urge to injure* some target” (Berkowitz & Harmon‐Jones, 2004, p. 108, italics added). Others characterize anger as “a *significant social problem* worthy of clinical attention and systematic research” (Beck & Fernandez, 1998, p. 63, italics added), while Waldman *et al*. (2011) generalize that anger expressions constitute a “problem” in the context of leadership studies.

We propose that these undifferentiated and negatively skewed perceptions of anger partly reflect what Stearns and Stearns (1985, p. 813) refer to as emotionologies, defined as “the attitudes or standards that a society … maintains toward basic emotions and their appropriate expression; ways that institutions reflect and encourage these attitudes in human conduct.” When scholars suggest that leader anger hinders attainment of their full (inspirational) leadership potential (Waldman et al., 2011) and psychologists “claim that they spend more time helping clients manage their anger than in dealing with any other emotion” (Kristjánsson, 2005, p. 679), anger appears to serve no redeeming individual nor societal function and should be, wherever possible, suppressed or eradicated. Further, of particular interest in the context of our article is the recent claim by management scholars that “*reducing* anger among employees is one potential avenue for *decreasing unethical* behavior in the workplace” (Motro, Ordonez, & Pittarello, 2014, p. 1, italics added).

For scholars concerned with the study of morality and justice, however, these assertions are likely to be unsatisfactory. Even researchers who argue that angry individuals (minimally) are “biased decision‐makers” acknowledge that “anger, even strong anger, is not inherently dysfunctional” (Novaco, 1994, p. 21). Thus, in our quest to better understand anger's *pro*social capabilities, we endorse Haidt's (2003) view that anger is likely the “most underappreciated moral emotion” (p. 856), and functionally—when *not* a lingering trait—more moral than immoral. Social functional accounts define emotions, and especially anger, in terms of consequences of goal‐directed behaviors (Keltner & Haidt, 1999). From this perspective, redressing injustice, for instance, is both a function and a consequence of anger.

In juxtaposing emotionologies with social functional accounts of anger, however, we see challenges for organizational theory, research, and practice alike. Specifically, varying anger definitions and imprecise differentiations between antisocial and prosocial aspects of anger contribute to the construct of moral anger remaining theoretically elusive and underdeveloped. Thus, the extant literature is in a state of disarray. Evidence of this is found in recent efforts to delineate the parameters of moral anger. For instance, the label “moral anger” is sometimes used as a key construct guiding empirical investigations without being defined in clear conceptual terms (Master, 2009). Frequently, moral anger is defined simply as anger provoked by perceptions that a moral standard—especially fairness or justice—was violated (Hoffman, 1989; Montada & Schneider, 1989). Elsewhere, the concept of moral anger can be so narrowly constrained for clinical experimentation as to limit practical application outside the laboratory (Batson et al., 2007).

We identify several issues here. First, the noted brief definition of moral anger does not capture processes associated with this prosocial form of anger in sufficient depth to allow a distinct conceptual construct to emerge. For instance, when does a violation appraisal occur and what action tendencies ensue that allow the phenomenon to remain “moral”? Critics argue further that such a simplified, undifferentiated definition can allow individuals to justify and apply the moniker of *moral anger* to anger that is only self‐serving (Batson et al., 2007; O'Mara, Jackson, Batson, & Gaertner, 2011). In addition, moral anger is often equated with moral outrage and conceptualized as part of a “moral hostility triad” (i.e., contempt, anger, and disgust or CAD) hypothesis (Rozin, Lowery, Imada, & Haidt, 1999), suggesting moral anger can have hostile intent (Gutierrez, Giner‐Sorolla, & Vasiljevic, 2012). Still others argue that expressed anger on behalf of unfair treatment toward a significant other does not fall under the category of moral anger, as it is *empathetic* toward others' harm (i.e., empathic anger; Batson et al., 2007). Given the degree of conceptual imprecision in the literature, subsequent experimental manipulations may have limited applicability. In summary, the existing confusion over what constitutes moral anger thwarts our ability to study—theoretically, empirically, and practically—anger's more socially functional, adaptive, and fairness‐enhancing components. This conceptual imprecision may also contribute to the promotion of ill‐informed organizational and societal policies and practice.

Part of the problem identifying what is moral anger stems from how scholars define the root emotion of anger. Scholars have cautioned on the lack of sufficient differentiation in terms of negative valence in emotion research in general (Gooty, Gavin, & Ashkanasy, 2009). Recent critiques of traditional (versus emerging) views of workplace anger acknowledge scholars' tendency to equate anger and its expression—even when appropriate under the circumstances—as a form of hostility and aggression, conflating the emotion of anger with both harmful intent and damaging consequences (Geddes & Stickney, 2011; Lindebaum & Jordan, 2014; Stickney & Geddes, 2014). Examples of this lack of differentiation between anger expression and aggression in terms of organizational practice and policy exist. For instance, the UK's National Health Service (NHS) initiated operational changes to address organizational perceptions of “problematic” behaviors among patients and visitors. These changes are reflected in recent advertising that advises patients to “choose their treatment” (i.e., medical personnel or police). 1This information has been retrieved from two relevant websites: (i) *New campaign to tackle violence against NHS staff* (2013) (Retrieved from http://www.wales.nhs.uk/sitesplus/955/news/26261, accessed on 20 October 2015) and (ii) *Your choice of treatment—Stop abuse of NHS staff* (Retrieved from http://www.healthpromotioninherts.nhs.uk/HPAC/MoreDetails.jsp?id=4679&dsn=hphertfordshire&subjectId=16&referrer=http://www.healthpromotioninherts.nhs.uk/HPAC/BrowseSearch.jsp?subjectId=16&typeId=P&submit=true&sort=dater&page=1, accessed on 20 October 2015). This threat visualized on prominent posters reflects a zero‐tolerance policy regarding patients or clients “verbally abusing” their staff (Welsh Ambulance Services NHS Trust, 2014). While abusive behavior should never be tolerated at work, the NHS unfortunately does not always differentiate *verbal abuse* from *verbal anger*. Thus, angry complaints regarding care (or lack thereof) from those concerned about their sick loved ones can potentially be labeled “assault and abuse,” potentially prompting sanctions including the threat of withholding treatment or being arrested (NHS, 2004, 2010).

Given concerns stemming from the previous example and issues raised earlier, the aim of this article is to articulate a theoretically relevant and practically expedient social–functional definition of moral anger, while conceptually delineating moral anger from other kinds of anger or anger‐related constructs that share an affective base. Moral anger, of necessity, implies inherently positive intentions (e.g., upholding moral standards, seeking fair treatment, and protecting those more vulnerable) and (ideally) corrective action yielding positive outcomes (e.g., moral standards are reinforced and vulnerable individuals are now better off). Nevertheless, while action readiness is embedded in the affective experience of moral anger, subsequent corrective behavior itself is distinct. Thus, we argue in this article that the act of blowing the whistle is but one possible behavioral manifestation following the experience of moral anger. Actions stemming from moral anger, through the sense‐making of individuals, can take other shapes and forms as well.

When the term “moral” is associated with a concept, however, issues of individual and cultural value systems, perspectives and varying standards regarding right and wrong can hinder general agreement and narrow possible ranges of moral behavior. Thus, we seek to identify specific characteristics that facilitate the highest possible degree of agreement for identifying moral anger. Further, the spirit in which we pursue this aim is couched in the tradition of “normative” theories, which—unlike the *Weltanschauung* of empiricists and rationalists—posit that *no* theory is value free (see Suddaby, 2014, for a detailed discussion). Instead, ethics and values of actors (as opposed to those of researchers) are appreciated in management theories as an engine that constructs reality, rather than a camera that captures it (MacKenzie, 2006). To borrow from Suddaby (2014), theory can serve various ends, and normative theory may offer its own genre of theoretical claims, including examples where normative elements (such as perceptions of right and wrong and resultant action) form part of a construct's definition. The key distinguishing feature of normative theories, as alluded to earlier, is that perceptions of right and wrong are reserved for actors based upon their ethical values, rather than pre‐imposed by researchers.

In this article, we discuss the following points. First, recognizing that moral anger is a unique construct in need of further clarification, we note distinguishing characteristics of moral emotions and specifically, moral anger, and illustrate why this construct can logically be differentiated as a prosocial form of anger. Second, we propose a detailed definition and elaborate on these characteristics to better distinguish moral anger from other related, but distinct, constructs (e.g., moral outrage and righteous anger), which share an affective base. Related to this is the goal of promoting greater consistency among scholars with regard to how moral anger is construed. Third, based on the proposed definition, we briefly provide examples of whistle‐blowing illustrative of how an individual acting out of moral anger could significantly improve the lives of others. Finally, we highlight how a greater sensitivity toward prosocial forms of anger can significantly improve organizational practice and future research.

## Conceptually Delineating Moral Anger

This section examines moral anger in the context of moral emotions and then demonstrates why it is useful and perhaps necessary to distinguish it from the emotion of anger itself. Later, we discuss our conceptualization of moral anger's unique qualities in relation to previous efforts to delineate its parameters and other relevant constructs.

### Moral emotions and anger

An assessment of morality requires that certain rules “are not the province of only a particular society … but, rather, rules which we apply to all people everywhere and expect them to obey” (Solomon, 1993a, p. 9). In consequence, moral rules are seen as universal, core to the moral fabric of society, and the driving force to do good and/or avoid doing bad (Kroll & Egan, 2004). Some argue that, owing to this universal character, they are disinterested and objective (Solomon, 1993a). This is consistent with deontological moralists, who focus primarily on motives of, for instance, angry actors. Kant argued that moral individuals do their ‘duty’ in pursuit of ‘good without qualification’ and out of respect for their fellow human beings (Solomon, 1993a), even though he also claimed that moral reasoning is ‘thoroughly objective.’ The moral individual is moved by a universal standard to fight injustice; there is no desire or intent to injure others, only to do or make right. Thus, moral anger would function as a tool to uphold acceptable standards of human conduct (Averill, 1982) and, concurrently, implies a lack of intentional harm.

Our conceptualization of moral anger also is generally consistent with Haidt's (2003) definition of moral emotions, as those emotions “that are linked to the interests or welfare either of society as a whole or *at least of persons other than the judge or agent*” (p. 853, italics added). Moral anger must be associated with something greater than individual self‐interest. 2Consistent with Solomon (1993a), we argue that drawing distinctions between disinterested and self‐interested elicitors of moral emotions, especially anger, can obscure more than it reveals. Solomon notes that “moral judgment is both the product of society and one of its constituent features. What we call our personal values are for the most part learned together and shared by a great many people” (p. 3). Taking this point forward, he cautions that the significance of choice in ethical debates is often mistaken with the view that individuals choose their values. This, he insists, is misleading, adding that “most moral choices involve decisions between already‐established possibilities and already‐available reasons” (p. 6). In other words, it is crucial to emphasize the intimate interplay between personal and societal values as we consider what makes us angry—and what functions this can serve. In other words, moral anger expressions would possess “greater good” instrumentality—where the self is not its prime or only beneficiary (Kelloway, Francis, Prosser, & Cameron, 2010). Thus, we argue that moral anger incorporates an outcome orientation of moral determination. That is, anger can be viewed as moral if others and/or social collectives are primary beneficiaries from its manifestation. This is also consistent with utilitarianism as espoused by John Stuart Mill (1861/2001). Moral anger would promote favorable outcomes for society and, ideally, be judged acceptable by the larger community (Jones, 1991). Thus, a hallmark of moral behavior and moral emotions is a primary concern for benefitting *others* (Haidt, 2003; Solomon, 1993a). This is well expressed in the so‐called Golden Rule (i.e., “Do unto others as you would have them do unto you,” Solomon, 1993). Both views of moral determination—motives (intentions) and consequences (outcomes)—are important components of moral anger, and both acknowledge that others' welfare and advantage is primary.

Concurring with this dominant perspective, we nevertheless recognize that the distinction between self and other interests does not always imply that to be moral, individuals must not personally benefit or should go against their own self‐interest. Along with Krishnamurti (1992) and Mill (1861/2001), we acknowledge that individual and societal benefits are often closely entwined. For instance, it might be possible that expressed anger based upon a personal affront can address a general moral issue in society. Thus, while we agree that one's interests can be addressed with moral anger, we nevertheless concur with established views that moral anger intentions and outcomes should largely benefit others.

Finally, if an individual experiences anger as a result of moral value violations, but does not express and act upon this emotion, then we cannot speak of moral anger. Moral anger cannot remain as a mere cognitive feeling; it necessarily prompts some form of expression and action. Rest's (1986) four‐component model for individual ethical behavior asserts that an agent must recognize the moral issue, make a moral judgment, resolve to place moral concerns ahead of other concerns (i.e., moral intent), and, importantly, act on the moral concerns. We attend to these aspects in the following sections.

### Moral anger versus anger

Earlier, we highlighted the tendency to equate anger with malfeasant intentions and/or destructive outcomes. For instance, “anger out” is cast as an act of someone who “engages in aggressive behavior when motivated by angry feelings” (Spielberger, Krasner, & Solomon, 1988, p. 95). However, we also find efforts to define displayed anger without a negative bias (Stickney & Geddes, 2014), including the dual threshold model's notion of “expressed anger” (Geddes & Callister, 2007) and the conceptualization offered by Gibson and Callister (2010). Their definition states that anger is “an emotion that involves an appraisal of responsibility for wrongdoing by another person or entity and often includes the goal of correcting the perceived wrong” (p. 68). This neutral characterization serves as an objective starting point for scholars and practitioners interested in the causes and consequences of anger in the workplace. However, if we contrast two very distinct scenarios, it is apparent this definition is less likely to capture the finer nuances of moral anger.

First, imagine struggling to find a parking space for an important professional appointment in the congested City Center. You are already late and intend to avoid further inconveniencing your colleague. Finally, you find a vacant parking spot and use the indicator (or turn signal) to show your goal to reverse the car into the vacant spot. However, another car drives into the vacant spot, despite your primary position and obvious intention to use the spot. Now you are both late and angry. A case like this falls into the category of goal obstruction as an elicitor of anger (Haidt, 2003). However, an appraisal of responsibility for a wrongdoing has occurred (i.e., he or she took my parking space!), and the ensuing anger experienced might prompt you to approach the culprit to correct the perceived wrongdoing (constructively or otherwise). We note that this situation aligns with Gibson and Callister's (2010) anger definition in terms of its appraisal processes and action tendencies. It too conforms to the definition of personal anger as espoused by Batson *et al*. (2007), who note its self‐oriented agenda and the potential for actions meant to punish others in more or less responsible ways.

By contrast, a study on the link between perceptions of unfairness and anger distinguishes itself from the aforementioned scenario. Raftopoulou and Lindebaum (2013) describe a situation onboard an aircraft (airborne), where the cabin crew wrongly accused an elderly lady of “pushing” a member of the cabin crew en route to the lavatory. Upon returning to her seat, the woman is approached by the cabin manager who sternly requests her booking reference and passport. She asserted that the woman had intentionally pushed a staff member and that the airline's policy did not tolerate such “rude” behaviors. Both authors—having witnessed the alleged contact—intervened as they believed the woman experienced seriously unfair treatment and intimidation by the cabin manager. The authors' intervention was motivated by the belief that nobody should be accused unfairly and that a stand for fair and respectful treatment was imperative, especially if violated by individuals with more formal power (i.e., the cabin crew; see Fast, Halevy, & Galinsky, 2012).

As in the first scenario, the parameters offered in the Gibson and Callister (2010) definition of anger apply to this scenario insofar as an appraisal of responsibility for a wrongdoing occurred (i.e., the authors attributed responsibility to the cabin crew for a violation of “fair” treatment and intervened to mitigate that perceived act of injustice and intimidation). However, the motivational force behind their action was not self‐interest, but a concern for others; this, in turn, placed them at some risk. 3We recognize that expressing anger on behalf of others can put an individual at risk, and this is a central argument in the literature on moral courage (Baumert, Halmburger, & Schmitt, 2013), which is why we include this notion in our definition of moral anger. In fact, they were threatened with police involvement upon arrival, although that did not actually occur. Contrasting these two scenarios should alert us minimally that different kinds of anger exists (Tavris, 1982). At the same time, they also illustrate why moral anger warrants a distinct conceptualization, for its defining features are clearly unique.

## Moral Anger (Re)Defined

Prior to offering a definition of moral anger, it is essential to draw attention to the technicalities of construct definition. Suddaby (2010) stresses that novel constructs typically are built upon, or constitute an extension of, extant constructs, as part of “an ongoing web of referential relationships” (p. 350). Among psychologists, this is typically referred to as the nomological net or network (Cronbach & Meehl, 1995). Thus, for any psychological construct to be of scientific utility, it must clearly capture variance, conceptually and operationally, in explaining outcome variables not accounted for by existing constructs (Brackett & Mayer, 2003).

In addition, Suddaby (2010) argues that, as part of offering construct definitions, scholars need to be careful not to conflate the processes that underlie a construct with its outcomes. In so doing, it has been argued that our own (i.e., as researchers) value judgments can predetermine the nature of the outcome (Lindebaum & Jordan, 2012). This is an important observation, and we concur with it in cases where scholars impose *their* value judgments. We noted this in our critique of emotionologies and definitions of anger that imply harmful intentions and consequences. However, in the tradition of normative theories, there is greater “focus on the motives and ethics *of actors* and the process by which they make choices for action” (Suddaby, 2014, p. 408, italics added), instead of value‐free causal logics. Therefore, we contend that it is plausible to incorporate both the processes associated with moral anger and its intended outcomes in our definition—as long as this puts the sense‐making processes of the angry actor center stage. Thus, having delineated fundamental characteristics of moral anger, we define it as

(i) an aroused emotional state stemming from (ii) a primary appraisal of a moral standard violation that (iii) impacts others more than oneself and (iv) prompts corrective behavior intended to improve the social condition, even in the face of significant personal risk.

Beyond capturing a more precise theoretical framework, this definition has other benefits. First, it offers flexibility in that it can pertain to one's own direct involvement as well as witness‐driven phenomena. Note that this does not, of necessity, detract from the other versus self‐interest distinction. As an individual, one may still be affected by violations of moral standards, but speaking up is undertaken with a view to defend others or the greater good, not just to facilitate one's own advantage. Consequently, moral anger is distinguishable from “personal anger” (i.e., anger where one's individual goals or interests are thwarted or harmed; Batson et al., 2007). Second, using simple present tense allows more flexibility in locating the violation and its consequences in the *now* and *future*. This is relevant as outcomes are often the driving force behind initiating appraisals, especially when primary justice appraisals are activated as undesired outcomes are experienced (Barclay et al., 2005). Because of the nature of violations of moral standards, there can be an additional projection into the future—in the spirit of “this should never happen again.” So one's own experience can be both in the present as well as in the future. The literature on anticipatory emotions offers further relevant insights. For instance, Baumeister *et al*. (2007) argue that, while emotions provide crucial feedback in relation to one's actions, the purpose or function of that feedback is to “learn a lesson” that provides guidance for future behavior. Third, it allows the inclusion of events other than those rooted in perceptions of injustice, although we argue that this is probably the most salient factor (Cahn, 1949). As a final emphasis, subsequent individual actions must attempt to correct the moral code violation, even if such efforts prove unsuccessful. This is because individuals operate (at work and beyond) under the influence of additional factors beyond their immediate control (Campbell, Dunnette, Lawler, & Weick, 1970). In the following sections, we expand upon each component of this definition with the intention of clarifying what moral anger *is*, and what it is *not*. Specially, we discuss moral anger in relation to a nomological network of related, but distinct, constructs (see Table 1).

**Table 1:**
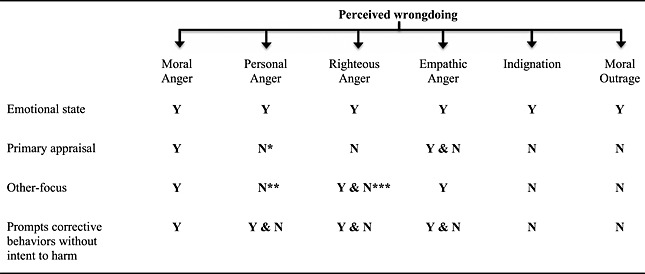
Conceptually delineating moral anger from related constructs

Y = yes; N = no.

* We suggest that moral anger and personal anger are distinguishable by way of different stages in the appraisal process. To recapitulate, we suggest that moral anger – based upon rapid automatic processing (Scherer, 1995) – indicates a reaction to the most pressing emotional challenges, including those of social‐evolutionary significance, as mentioned (see Gibson and Callister, 2010). In contrast, personal anger is based upon “a secondary appraisal of relevance, attributions, possible outcomes, goals” (Gibson and Callister, 2010, p. 71). Consistent with appraisal theories of emotions, secondary appraisals involve more complex meanings and analyses in relation to, for instance, perceived goal obstruction, consistency with norms or social standards, and fairness (Smith & Ellsworth, 1985).

** Our reading of Gibson and Callister (2010) and Batson et al. (2007) suggests that personal anger often resides in the context of self‐serving interests in relation to ‘offense’ or ‘goal interference’. However, Gibson and Callister (2010) also note “that not only will individuals react with anger when they are treated unfairly, they will also feel anger . . . when they perceive that the treatment of others, even strangers, is unfair” (p. 70).

*** The work of Tripp and Bies (2010) allows for revenge (as motivated by righteous anger) to serve both self and other interests. They note that “while the act of revenge may have served self‐interest, it often serves other interests, and it is usually justified in moral terms” (p. 428).

### Emotional state

As Frijda (2007) claims, all emotions are potentially adaptive states of action readiness. To better understand moral anger, the first point of differentiation is to clarify this construct as state (versus trait) anger. Moral anger is unlikely an emotional response rooted in trait anger, albeit at this stage we cannot say conclusively that it *never* can be. Trait anger is a stable although typically undesirable individual disposition (Domagalski & Steelman, 2005). Those with high trait anger report anger episodes that are more frequent, intense, and longer‐lasting than those with low trait anger (Tafrate, Kassinove, & Dundin, 2002). Further, this personality trait is often associated with hostility, verbal abuse, leader or petty “tyranny,” and aggression (Bettencourt, Talley, Benjamin, & Valentine, 2006; Domagalski & Steelman, 2005; Kant, Skogstad, Torsheim, & Einarsen, 2013). In such a case, anger is associated with highly self‐interested appraisals and antisocial action readiness (Haidt, 2003). Seen in this light, subsequently expressed anger often constitutes a deficiency. Based upon preceding sections, we maintain that consequences associated with trait anger are likely key drivers for negatively skewed perceptions of anger.

In contrast, moral anger is a form of state anger, an emotional condition that emerges in response to situation‐specific elicitors (Gibson & Callister, 2010). One's emotional state of arousal may range from more mild to more extreme levels of intensity. However, it is probable that higher levels of felt anger intensity would promote a response to a perceived moral violation, given that intensity of feeling helps overcome inhibitions to suppress or hide one's anger (Stickney & Geddes, 2014). State anger is a temporary (rather than stable) human condition. Like all emotions, moral anger is an individual's heightened cognitive, psychological, and physiological response to specific stimuli that remain salient for a particular period of time (Zeelenberg, Nelissen, Breugelmans, & Pieters, 2008), often until the situation is resolved. Unlike moods that have more vague triggers and longer durations, emotions emerge quickly as we notice and evaluate a particular, relevant change in our social environment (Solomon, 1993b).

An important caveat needs to be added in relation to how emotion traits and states can interact. According to trait–state theory (e.g., Deffenbacher et al., 1996), individuals high in a given trait are also more likely than those low in that trait to exhibit the state form. Conceivably, individuals high in trait anger would be generally more likely to experience state anger as well. Nevertheless, we argue that moral anger would necessitate that one's situational anger be based on circumstances negatively impacting or harming others, not oneself. Thus, it is unlikely trait anger is correlated with moral anger.

### Primary appraisal of moral standard violation

Frequently articulated elicitors of anger include injustice and unfairness, along with issues of interpersonal conflict and goal obstruction (Geddes & Callister, 2007; Horberg, Oveis, & Keltner, 2011; Van Kleef & Côté, 2007). We maintain, however, that moral anger reflects higher‐order elicitors and a concomitant awareness and belief in values and rules associated with civil society. 4We borrow the notion of “higher order” from Mill (1861/2001). He applies this notion to human pleasures to show that the pursuit of higher‐order pleasures (e.g., cultivation of an educated mind and moral sentiments) bears lower risks upon society as does the pursuit of lower‐order pleasures (i.e., those rooted in mere bodily sensation, such as personal indulgences), for the former comes with “greater permanence, safety, and uncostliness” (p. 15). It emerges when an intolerable situation accrues from violations of a moral standard, as opposed to reacting to mere goal obstruction or personal offense. Consequently, moral anger is seen as a “counterfactual” emotion in that it arises when an actual state of affairs is compared with more favorable and possible conditions (cf. Salovey & Rodin, 1985).

We anticipate moral anger to result from primary (versus secondary) appraisals of arousal, triggered immediately by offensive or unjust external phenomena. Novaco (1986) sees the cognitive mediation in anger “as an *automatic* and intrinsic part of the perceptual process” (p. 14, italics added). Cahn (1949) argued one's sense of injustice is “the *sympathetic reaction* of outrage, horror, shock, resentment, and anger, those affections of the viscera and abnormal secretions of the adrenals that prepare the human animal to resist attack” and added that “nature has thus equipped all men to regard injustice to another as *personal* aggression” (p. 24, italics added). Further to this, Master (2009, p. 103) found in her qualitative study on moral anger and social activism that her participants possessed an “intrinsic knowing” inasmuch as they “simply *knew* something was wrong,” in relation to, for instance, racial attitudes.

As opposed to advanced cognitive appraisals, prior studies suggest that affective reactions (which can be non‐conscious and automatic; Van Kleef, 2014) are primary when expectancy violations occur. To elaborate, experimental studies show that negative (but not positive) expectancy violations are a key determinant of affective reactions, occurring immediately following exposure to stimuli. Bartholow *et al*. (2001) argue that social perceivers tend to automatically evaluate other individuals and their behavior, including whether or not individuals conform to social stereotypes. These affective reactions may also help explain why individuals perform better at identifying misbehavior of transgressors (i.e., a social actor/agent to whom perceptions of wrongdoing are attributed, see Folger, Cropanzano, & Goldman, 2005) than other kinds of cognitive tasks. Le Doux (1996) argues that there is “incontrovertible evidence that affective reactions could take place in the absence of conscious awareness of the stimuli” (pp. 54–55). Automatic reactions in response to violations of moral codes are also linked to evolutionary advantages; here, anger is seen as a signal telling us that important moral values are being violated (see Gibson & Callister, 2010, for a short discussion). For this reason, we propose that moral anger emerges from a primary appraisal of stimuli as opposed to more deliberative, secondary cognitive appraisals. In other words, we argue that moral anger's primary appraisal is a “gut reaction” and can occur without conscious processing.

Examples of more deliberative appraisals can be found in the related constructs of indignation and righteous anger, even anger itself. On the one hand, indignation represents an advanced cycle of the appraisal system, occurring subsequent to appraisal processes of deliberation, negligence, or avoidability at the schematic meaning level (Power & Dalgleish, 2007). Also considered a “morally grounded form of anger” (Jasper, 2014, p. 208), indignation tends to reflect a more self‐oriented focus with corresponding idiosyncratic standards (Solomon, 1993b). Rather than a general sense of something amiss, there is a more conscious determination of personal affront. Moreover, in the words of Sims (2005, p. 1627), indignation entails a “conviction of one's own rightness and the other's wickedness.” In the context of work (and beyond), he also suggests different action tendencies, such as expressing indignation through humor, while others might express it as it is felt, thereby not avoiding conflict. Overall, as Haidt (2003) notes, even though anger and indignation belong to the same “emotion family” (he considers the latter the “child” of the former), they are not identical in their eliciting conditions and action tendencies.

The construct of righteous anger, defined as an “emotional response to correct and prevent injustice” (Tripp & Bies, 2010, p. 413), is part of a growing body of research on *revenge as justice* (also McLean‐Parks, 1997; Tripp & Bies, 1997). Specifically, Tripp and Bies (2010) suggest that this reflects motivations “for revenge … grounded in a perception that one has been the victim of *undeserved* harm and feelings of injustice” (p. 416, italics in original). Righteous anger emerges when offended individuals see themselves as victims of undeserved harm and injustice and are further motivated to use actions, such as revenge, to correct the injustice (Tripp & Bies, 2010). It too is described as an intentional and directed response toward perceptions of harm or wrongdoing (Tripp & Bies, 2010). However, righteous anger is not necessarily constructive and prosocial but depends on who is getting angry, what they do about it, and who is telling the story (Potegal & Novaco, 2010). Thus, this type of response may reflect self‐interested action tendencies, requiring higher‐order cognitive reflections (e.g., attributing blame; Berkowitz, 1990) versus automated or unconscious appraisals. Thus, a clear demarcation emerges between moral anger and other related constructs. Whereas secondary appraisals normally pertain to interpreting and ascribing meaning to an event, moral anger as a primary appraisal more typically reflects the salience of an event to social well‐being and fundamental moral values (Smith & Ellsworth, 1985).

### Focus primarily on others, not self‐interests

This distinguishing characteristic of moral anger was introduced in our earlier discussion of what constitutes “moral” emotions. Key for moral anger is that one's anger, expression, and response are based more on concern for others than oneself. To emphasize this point, we distinguish moral anger's primarily other‐oriented focus with that of righteous anger, indignation, and personal and empathic anger. The previous section indicated that righteous anger can be a catalyst for individual action that is self‐protective and retaliatory in nature, in order to “even the scales of justice” (Weiner, 2005, p. 37). Individuals see themselves as victims who are justified to pursue revengeful actions. With indignation, subsequent anger reflects personal offense and pride, prompting feelings of contempt toward the offending party. Solomon (1993b) indicates that *self*‐righteousness occurs when individuals become convinced of another's “objective” guilt. This often leads the offending person and his or her behavior being viewed as inferior or wrong while the offended, angry party consider themselves superior and their response “right.” As a life strategy and consistent with fundamental attribution errors, individuals may avoid acknowledging personal weakness and vulnerabilities and instead attend to the moral frailties of others (Solomon, 1993b).

Again, while moral anger, righteous anger, and indignation have some overlap, they differ with regard to the focus of their primary interest and concern. Although angry individuals may benefit from their moral anger, their salient concern is a broader benefit to others, rather than limited to self‐interest. Geddes and Callister (2007) refer to this attribute of what observers would classify “appropriate” anger as reflecting an alter‐centric versus ego‐centric base. Specifically, when individuals can demonstrate their anger is tied to offenses impacting others, more than themselves, observers of anger displays are more inclined to view expressed anger as acceptable in an organizational setting. Further, Geddes and Callister (2007) also emphasize the notion of anger advocacy. Here, angry employees approach supportive confidants who then act as advisors, coaches, or even personal advocates on behalf of their colleagues to help address problematic work situations. In these instances, they argue that organizational members “who act as advisors, advocates, and/or surrogates … increase the probability of more positive than negative outcomes” [in the workplace] (p. 728).

Research attempting to distinguish moral anger or moral outrage (sometimes used interchangeably in the literature) from personal anger clearly shows that the latter emerges “when one's own interests are thwarted … or one is treated unfairly” (Batson et al., 2007, p. 1273; but see also O'Mara et al., 2011). Unfortunately, individuals attempt to label this (by definition) self‐serving, personal anger as a moral emotion in order to gain legitimacy and perhaps supporters. However, often the goal is less about restoring fairness and more about protecting one's own interests and, in some cases, seeking revenge or restoring one's honor (Cohen, Nisbett, Bowdle, & Schwarz, 1996). In contrast to personal anger, differentiating moral anger from empathic anger appears, at least initially, less clear‐cut. Batson *et al*. (2007) introduced the concept of empathic anger as the emotion felt when the interests of a “cared‐for” other are thwarted. Conceivably, because one is not personally impacted, this could reflect the “focus on others” requirement for moral anger. However, it is argued that such empathetic responses are not based on an assessment of a moral standard violation as much as simply a significant others' own interests (Batson et al., 2007). While we would argue strongly that felt empathy and/or compassion, regardless of beneficiary, likely affects one's ability to experience moral anger (Hoffman, 1989; Montada & Schneider, 1989), in its current conceptualization as proposed by Batson *et al*. (2007), empathic anger is seen less as a response to fairness standards violation, and more focused on acknowledging and addressing harm caused to someone with whom one is affiliated.

### Prompts corrective behavior intended to improve the social condition

It has been argued that the two most widely studied types of social behavior are helping and harming behavior (Lee & Allen, 2002). Logically, organizations are better off when helping is optimized and harming is minimized. Moral anger, as defined here, serves to avoid harm while improving upon or removing a fundamentally unacceptable situation. Thus, prompting helping behavior, moral anger attempts to reconcile disparity, repair damaging situations, restore equity, and, in general, improve the human condition. In this respect, Adams (1995) shares the following observation:

If anger is not guided by the optimism of vision and clear humanistic values, it can be diverted into desperate and anti‐human activities. The enemies of peace and justice often try to exploit anger in order to divert movements into such desperation (p. 17).

Important to the anger/aggression distinction reinforced in this article, Adams is also explicit that anger, thus conceived, “is not the same as violence” (p. 18). His observation is both consistent with, and lends further substance to, our definition of moral anger. However, there is a large body of research focused on behavior meant to harm, such as aggression (Baron & Richardson, 2004). Even indignation has been associated with a desire “to punish” (Solomon, 1993b, p. 271). To elaborate, aggression is defined as “any form of behavior directed toward the goal of harming or injuring another living being who is motivated to avoid such feeling” (Baron & Richardson, 2004, p. 7). In the context of the workplace, aggressive behaviors are said to take three distinct manifestations: (i) overt aggression (e.g., physical assault or violence); (ii) verbal and/or symbolic expressions of hostility (i.e., verbal abuse or gestures); and (iii) obstructionism (e.g., withholding efforts or information to impede progress of others; Skarlicki, Folger, & Tesluk, 1999). Baron and Richardson (2004) argue that, while emotions and attitude *can* play a role in the occurrence of behavior that causes harm, their presence as such is not a precondition for the enacting of such behaviors.

In relation to moral outrage, while it is also said to be a “potent source of moral motivation, prompting efforts to restore fairness and justice,” the subsequent reference to achieving this “either by compensating the victim or by punishing the harm‐doer” (Batson et al., 2007, p. 1272) sets it apart from moral anger advocated here in two meaningful ways. First, compensation implies that a moral transgression is accepted as a given and that ways are found to make the situation more tolerable for the victim. Moral anger, by contrast, seeks to correct the moral transgression here and now as well as in future, as explained earlier. Second, moral anger lacks the desire for intentional harm (e.g., punishment and vengeance) toward the harm‐doer. Moral outrage, by contrast, implies a high level of intensity (i.e., “rage,” which suggests more potential to damage or harm). In summary, given that all these anger‐related constructs have an inherent “intent to harm” motivation, none can be construed as moral anger.

Our conceptualization of moral anger proposes that one's intentions behind the action to correct a moral violation—not one's actual (or perceived) success—constitutes moral anger's connection to subsequent action. Determining whether someone's actions actually succeed in correcting moral violations is not always straightforward due to variations in individuals' judgments and perceptions. However, one potential way to assess the relative “success” of moral anger expression comes from the work of Near and Miceli (1995) on effective whistle‐blowing and associated criteria to gauge the “reasonableness” of its consequences. In other words, did whistle‐blowing lead to more favorable outcomes for others who had previously been negatively impacted; that is, are people better off as a result of this action? Further, it seems reasonable that the more beneficiaries there are, the more we can speak of expressions of anger as morally and socially functional. For instance, did the act of whistle‐blowing—as motivated by moral anger—initiate changes in relevant legislation, so that, for example, relevant authorities exercise closer control over medical trials leading to the approval and use of new medical drugs? As the recent case of a pharmaceutical contract research organization shows, their fabricated drug test results potentially put the lives of many patients at risk. Across Europe, several relevant authorities have suspended marketing approval over concerns about clinical trials conducted by the contract research organization as a result of the whistle‐blower's actions (Burger, 2014). In this case, at‐risk populations globally appear to have benefitted substantially. In another example, public opinion that once cast an individual or a small group of individuals as “emotional deviants” (Thoits, 2004) now has come to terms that these individuals—due to their perceptions of injustice—played a crucial role in the transformation of others into necessary “counter‐normative peer groups” to help initiate the redressing of injustices (Thoits, 2004). For instance, the activist and retired professor Angela Davis, once branded a “terrorist” by President Nixon, is now viewed as a moral authority on racial discrimination (Jeffries, 2014).

In summary, having defined and provided detailed background on moral anger, we propose that moral anger is potentially a cumulative phenomenon in the sense that some anger expressions may be viewed as more moral than others. The more anger expressions reflect the four characteristics discussed previously, the more likely it can be viewed as *moral* anger. We illustrate the conceptual delineation of moral anger from other anger‐related constructs in Table 1. Note that perceptions of wrongdoing mark the starting point for each construct, with the four distinguishing characteristics of our moral anger definition revealing distinct variations among the concepts under review. In the following section, we apply our definition of moral anger to the literature on whistle‐blowing—as one type of behavior prompted by moral anger—to highlight its conceptual utility.

## Examining whistle‐blowing as a consequence of morally motivated anger

The whistle‐blowing literature offers manifold examples to which we can pertinently apply the lens of moral anger to either predict different courses of action (e.g., other than those predicted by aggression or simple inaction) or as a verifying tool to show that morally angry individuals can enact prosocial behaviors consistent with our conceptualization of moral anger to improve a given situation. Whistle‐blowing has been defined as “the disclosure by organization members (former or current) of illegal, immoral, or illegitimate practices under the control of their employers, to persons or organizations that may be able to effect action” (Near & Miceli, 1995, p. 680). The act of whistle‐blowing is most often an act that facilitates exposure of illegal, unethical, or morally questionable acts that may go against the values of the organization as well as civil society (Miceli & Near, 1988). We offer three brief examples of whistle‐blowing to illustrate where an individual's moral anger (i) might have prevented human tragedy, (ii) may or may not produce sufficient beneficial societal outcomes to ultimately warrant this designation, and (iii) identified unethical practices that changed an industry.

In the presence of moral anger, we envisage the following case to potentially have taken a different course of action. Specifically, consider the radiation experiments secretly undertaken by the U.S. Department of Defense on both soldiers and civilians during the late 1940s and early 1950s (Nielsen, 1996). These experiments exposed humans to varying degrees of radiation, and often without obtaining consent or informing research subjects on the significant risks involved. While there was considerable debate and opposition against these experiments among the scientists involved, several of them “obeyed orders, cooperated, and did not blow the whistle” (Nielsen, 1996, p. 16). This was, in large part, because the Department of Defense classified the relevant documentation on and around the experiments as “secret” to avoid adverse public debates and potential lawsuits. At that moment in time, the scientists were driven by fear of penalties for breaching the military's confidential classification. Thinking the risk of whistle‐blowing was too high, the scientists' decision not to pursue the issue allowed the unethical experiments to continue until 1953.

As a more contemporary example, consider the still‐pending case of Edward Snowden. There is considerable divergence in public opinion as to whether it was right or wrong to blow the whistle on the (for him, unlawful) surveillance activities of the U.S. intelligence services. However, commentators suggest that his anger (Holings, interviewed by Hinson, 2013) stemmed from the perception of unjust and illegal governmental practices. More specifically, some note that “behind his quiet, unassuming surface, his … *anger* with his employers was growing” (Harding, 2013, cited in Moretti, 2014, pp. 845–846, italics added). Further to this, in a letter to the German MP Christian Ströbele dated 31 October 2013, Snowden asserts he was compelled out of a “moral duty” to act against “systematic violations of law” and that his concern was geared toward “upholding the international laws that protect us all.” 5The letter can be retrieved from the BBC website. Please follow this link: http://news.bbc.co.uk/1/shared/bsp/hi/pdfs/snowden‐brief100%5B1%5D.pdf, accessed on 20 October 2015. Should this link not be operational in the future, please request a copy from the first author. As we noted in our definition of moral anger, one's actions must be viewed as ultimately beneficial to society in general, possibly putting oneself at some risk, to be classified as moral anger, and to date, Snowden's personal account and current status reflect this.

Perhaps less contentious are examples of corporate whistle‐blowing, such as the infamous case of U.S. Tobacco. This celebrated instance clearly shows that individuals appalled by corporate practices can enact prosocial behaviors consistent with our conceptualization of moral anger. Nevertheless, selfless actions meant to help society may require significant risks—both professionally and personally. For instance, in 1996, Jeff Wigand, then Vice President of Research and Development at Brown and Williamson Tobacco and responsible for developing reduced‐harm cigarettes, blew the whistle that his company intentionally increased the amount of nicotine in their cigarettes, thereby enhancing its addictive nature. As part of his (initially) internal challenges, he reported being harassed and receiving anonymous death threats. His situation was portrayed in the 1999 film, *The Insider*. In a 1996 interview by Mike Wallace of CBS 60 Minutes, Wigand stated that he “got angry” about the company's decision to abandon the safer cigarette, although he did not blow the whistle at that time. 6The full transcript has been retrieved from http://www.mcspotlight.org/beyond/cbstranscript.html, accessed on 20 October 2015. Once he did find the courage to speak up, his whistle‐blowing brought significant danger to him and his family but also contributed to the exposure of unethical practices by U.S. tobacco companies. Ultimately, Wigand's actions benefitted potentially millions who would have been exposed to this especially harmful but legal product.

In light of these examples, we concur with Tavris (1982), who posits that there may be many mundane (or extraordinary) incidents when individuals *angrily* take a stand for what they deem is right at work and in a democratic, just society. In so arguing, we recognize that moral anger is always expressed (versus suppressed) anger that may be displayed in the form of facial expressions, tone of voice, and non‐harming behaviors. More broadly, we recognize that moral anger would energize (cf. Lindebaum & Gabriel, 2015) individuals to act in restorative ways for the betterment of others (i.e., coworkers, clients, and communities) adversely impacted by violations of moral codes, even though the entity or person responsible may never be faced personally. Regardless of one's success, moral anger reflects one's desire to speak up and do something on behalf of others and for the betterment of civil society and their respective organizations.

Our examples alert us to what can happen in the absence and presence of moral anger. If we apply our conceptualization of moral anger, it only takes one courageous individual to blow the whistle and help generate public momentum that contributes to the cessation of illegal and unethical practices—many of which, if allowed to continue, could lead to human tragedy. Of course, the personal risk involved in disclosing information that an organization wants desperately to remain private has undoubtedly contributed to “many good [and angry] people doing nothing,” to borrow sentiments from Edmund Burke (Bromwich, 2014, pp. 175–176). We are nevertheless encouraged that in the U.S.A. and other countries (e.g., the U.K.), relevant legislation encouraging and protecting whistleblowers has advanced significantly in recent years. Examining whistle‐blowing as an example of moral anger helps us better understand how perceptions of injustice and violations of social norms motivate behavior seen as improving the social condition, even in the face of personal risk.

## Future Research on Moral Anger

We have proposed that moral anger is an explicit and distinct construct whose conceptual parameters are in need of clearer delineation. As such, we have maintained this refined construct has significant potential to enrich future theorizing, empirical testing, and organizational practice. Having laid theoretical groundwork, we recognize the need to pursue ways to operationalize or investigate moral anger in organizations (Cooper, 1982). With this goal, we envisage at least three avenues.

First, for researchers favoring the laboratory setting as a means of gathering data, it would be useful to design specific vignettes (or emails, e.g., Johnson & Connelly, 2014), whose wording is unambiguously and consistently guided by, and rooted in, the proposed definition of moral anger. A second approach to operationalize moral anger is the design of a specific psychometric instrument to assess self or others' perceptions of moral anger (depending on the exact aim of a study). Authoritative reviews on the topic are well established (Clark & Watson, 1995; Worthington & Whittaker, 2006). As a starting point, in our definition, we propose four key factors of moral anger for which researchers could develop preliminary items: (i) an aroused emotional state; (ii) primary appraisal of moral standard violation; (iii) impacting others more than self; and (iv) prompting corrective behaviors intended to improve the social condition, even with the possibility of significant personal risk. Further, we suggest moral anger could be distinguished as a “cumulative effect” of all four variables, whereas other examples of prosocial or morally motivated anger (e.g., empathic anger or moral outrage) would reflect fewer characteristics (Table 1).

As a novel construct, moral anger must also demonstrate construct validity. Anastasi and Urbina (1997) describe construct validation as a process of evaluating whether a given test actually measures some theoretical construct or trait. Such process seeks, *inter alia*, to determine whether the instrument assesses empirically what it purports to measure theoretically and employs statistics such as exploratory (and, ultimately, confirmatory) factor analysis (Huck, 2004). Table 1 should help establish discriminant and convergent validity (Campbell & Fiske, 1959) as we attempt to differentiate moral anger from other distinct, anger‐related constructs (i.e., personal anger, righteous anger, empathic anger, indignation, and moral outrage). To this end, consistent use of terminologies is required. Relatedly, although even the most precise measure of moral anger will, of necessity, show some overlap with measures of other forms of anger given their shared affective base, we recognize that underlying common variance should not detract from establishing discriminate validity. Therefore, we do not know yet whether, at this formative stage, traditional exploratory and confirmatory factor analyses are effective in establishing discriminant validity. We recognize the possibility that many of the common “rules of thumb” in relation to cross‐loadings and model fit that are applied to these analyses might indicate a lack of discriminant validity among the different forms of anger—no matter how well‐designed the measures are. Therefore, we suggest that this can signal a limitation of the analytical procedures, but not of the construct itself. In such a case, we recommend either to relax the aforementioned rules of thumb or to focus upon incremental predictions of moral anger. For instance, for moral anger to qualify as a novel construct, it must explain variance in important work or life outcomes not accounted for by existing constructs. At a minimum, we argue that moral anger could be used to examine prosocial forms of anger that promote urgently needed change, enhanced dialogues and discussion, and challenges to unethical and illegal practices. Other potential dependent variables around which specific propositions could be articulated include engagement in challenging authority or the status quo, prosocial dissent, whistle‐blowing, social activism, advocacy, disposition to defend free speech, altruistic behaviors that carry personal risks, and acting against inequalities in access to justice or resources.

Finally, we propose that the definition of moral anger provided here can also serve qualitatively inclined scholars as a guide to structure and formulate interview questions. This may be of particular interest in cases when and if, as hinted earlier, statistical analyses fail to delineate moral anger from other anger‐related constructs. Again, this may not be an issue with the construct as such but rooted in the limitations of the statistical procedure. Recent studies show how insightful the eliciting of lived experiences in relation to anger in the workplace can be (Lindebaum, Jordan, & Morris, 2015).

As ongoing scholarship further validates or investigates this construct, we envision various studies that could be pursued. For example, given the significant number of studies linking trait anger and negative affectivity with unfavorable, anger‐related workplace outcomes (Thoresen, Kaplan, Barsky, Warren, & de Chermont, 2003), more research examining moral anger and positive workplace outcomes could examine moral intensity and individual traits such as positive affectivity (Watson, Clark, & Tellegen, 1988). For instance, it has been proposed that an issue's “moral intensity” can promote a higher recognition of ethical issues and ethical behavior (Master, 2009). It might be that moral intensity moderates occurrences of moral anger at work or in society, in general. Some initial research on expressed anger also suggests that an individual's positive affectivity, more than negative affectivity, determines whether or not individuals express anger at injustices and to individuals best suited to address the problem (Stickney & Geddes, 2014).

Service and work relationships that may include client anger expressions are additional opportunities to examine moral anger, for instance, in relation to “communal motivation” (i.e., the degree an individual wishes to be responsive to a relationship partner and assume responsibility for their welfare; Mills, Clark, Ford, & Johnson, 2004). For example, Yoo *et al*. (2011) found that individuals classified as having “low communal motivation” judge angry partners more negatively, provide lower support, and have lower relationship satisfaction compared with those with high communal motivation. Applied to service providers in the healthcare industry, for instance, those (e.g., nurses) most interested in patient well‐being might have more positive reactions to their expressed emotions (i.e., they would be less likely to view anger expression by patients, for example, as abusive) and be more inclined to withhold offense or negative judgment to better understand and address client needs. Thus, we anticipate that employees with high communal motivation and/or positive affectivity might be more able to distinguish justifiable and moral anger and be more likely to exhibit it themselves when confronted with organizational injustices and improprieties.

We also envision that a range of concepts, such as optimism, empathy, altruism, and courage, could be examined in relation to moral anger as a relevant phenomenon that can promote more supportive, just, and honorable work environments. Future research also could explore the frequency and timing of when the effects of moral anger expression in the context of work manifest themselves. Importantly, we stress that even a single occurrence of moral anger can make all the difference in redressing acts of injustice. This features prominently in the literature on social movement (e.g., Jasper, 1998, 2014), alerting us to the fact that individual moral anger can also morph into a group‐level phenomenon. These examples of future research are provided to reiterate that we see moral anger as a unique, prosocial component in the wider nomological network of anger and anger‐related constructs. At this juncture, however, we recognize that this article simply lays the groundwork for further, significant research aimed at conceptual refinement and practical application.

## Recognizing Justifiable and Prosocial Forms of Anger in Organizations

Ghoshal (2005) once observed that “since morality … is inseparable from human intentionality, a precondition for making business studies a science has been the denial of any moral … consideration in our theories and, therefore, in our prescriptions for management practise” (p. 77). We posit that the acuity of his observation has grown in significance in recent years as a result of emotionologies that increasingly cast organizational anger expression as deviant, harmful behavior (Lindebaum & Jordan, 2014). As a consequence, organizational efforts to eliminate whistle‐blowing as well as other forms of appropriate, morally justifiable emotional expression at work can in truth conceal unethical and unjust practices that put individuals, organizations, or the environment at risk. Having argued that prosocial forms of anger, including moral anger, serve important roles in maintaining civil society, we come full circle back to the proposition that allowing morally motivated anger expression in organizations can serve as a tool of organizational diagnosis to better our individual and collective behaviors (Lindebaum & Gabriel, 2015), including more just treatment of relevant stakeholders and populations.

Let us briefly revisit the case of the NHS in the U.K. We offer this analysis as a cautionary tale of potential problems in decision making and policy formation when organizations do not differentiate justified and even morally motivated anger expression from verbal assault and abuse. Simply put, workplace anger displays often signal problematic situations (including policies, employees, or conditions) that need attention in order to improve customer/patient service and confidence in the organization and its staff. Confusing morally motivated anger with verbal assault or abuse can stigmatize and invariably suppress important information about workplace conditions and treatment that could, in reality, benefit future services, operations, and patients. For instance, when the NHS asked staff to report *non‐physical assault*, they provided two categories: “verbal abuse, excluding verbal threats of physical violence” and “verbal threats of physical violence” (NHS, 2010, p. 91, see footnote no. 12). Asking survey respondents to identify verbal threats of physical violence is fairly clear and straightforward. However, the term, “verbal abuse” is used increasingly by the lay community to describe any intense or intrusive verbal action one would *prefer not* to experience, rather than its actual definition of demeaning, injurious, and untrue statements meant to cause emotional and psychological distress. Interestingly, primary causes of “verbal abuse” listed by NHS staff overwhelmingly included the patient's own mental health condition. Related causes of verbal or physical abuse (*undifferentiated* in a second study report; NHS, 2010) reported by staff included numerous factors related to NHS information, operations, and facilities, including the following: (i) length of time waiting to be seen by a health professional; (ii) problems understanding information/instructions; (iii) dissatisfaction with services/treatment; (iv) concern about their condition; and (v) concern about another patient's condition (NHS, 2010).

Overall, with the NHS example, our contention relevant to moral anger and all forms of prosocial anger is twofold. First, failing to differentiate anger expressions from verbal abuse and assault can reinforce the inaccurate, although common belief that all displayed anger is hostile, aggressive, and violent; in other words, all anger is viewed as having no social or redeeming value in organizational settings. Second, often, one's anger is clearly justifiable. Thus, when a family member advocates vehemently on behalf of their suffering or mistreated relative, it can function as a diagnostic tool to identify faults, limitations, and weaknesses in organizational processes and employee practices. Consequently, information gleamed from angry clients and their families can promote greater dignity, assistance, and kindness toward a particularly vulnerable population. 7We have retrieved the above NHS information from publicly available sources. To what extent NHS staff actually follow or enact these policies, or whether all NHS staff are aware of these policies, is something we cannot establish here.


For this possibility of better service and functioning, however, organizations need to widen (not compress) their “zone of expressive tolerance” (Fineman, 1993). This increased individual and organizational degree of freedom for emotion‐triggered communication practices can create a greater civic space for ethics to operate (Ricoeur, 1991), where employees attempt to better understand and address the source of anger at injustice or indignities (i.e., moral anger versus simple goal frustration) and to engage constructively in conflict resolution required to create a better situation than before. We are both encouraged and sobered by recent studies showing that expressed anger predicts perceived improvement with problematic situations at work, while suppressed anger induced perceptions that the situation at work deteriorated (Stickney & Geddes, 2014). Thus, sometimes, it can be *good to feel bad* at work (Lindebaum & Jordan, 2014), especially if that means allowing client anger displays that address inappropriate, unjust, or disrespectful actions or practices.

## Conclusion

In this article, we challenge ongoing beliefs in society and organizations alike that anger is *always* bad, deviant, and even dangerous. Instead, we assert that anger has important prosocial forms that need to be better understood and, at least, allowed because they help us identify and address problematic situations in work and beyond. In particular, we offer an explicit and refined conceptual definition of moral anger and delineate various types of anger‐related constructs to better distinguish this “highest form” of socially functional anger. We address the current confusion and disarray over what constitutes moral anger and suggest this limits the way we theorize about and advocate for this type of anger at work and in society. Our hope is that future theoretical, empirical, and practical efforts to examine and incorporate moral anger in the broader domain of psychology and management studies can recognize its vital role in sustaining just and fair workplaces and societies.
